# The Development of a Selective Colorimetric Sensor for Cu^2+^ and Zn^2+^ in Mineral Supplement with Application of a Smartphone Paper-Based Assay of Cu^2+^ in Water Samples

**DOI:** 10.3390/s24237844

**Published:** 2024-12-08

**Authors:** Mahmoud El-Maghrabey, Shōta Seino, Naoya Kishikawa, Naotaka Kuroda

**Affiliations:** 1 Graduate School of Biomedical Sciences, Course of Pharmaceutical Sciences, Nagasaki University, 1-14 Bunkyo-machi, Nagasaki 852-8521, Japan; dr_m_hamed@mans.edu.eg (M.E.-M.); ssbasukeboy@yahoo.co.jp (S.S.); n-kuro@nagasaki-u.ac.jp (N.K.); 2 Pharmaceutical Analytical Chemistry Department, Faculty of Pharmacy, Mansoura University, Mansoura 35516, Egypt

**Keywords:** copper(II), zinc(II), NBD-Cl, Girard’s Reagent P, paper-based assay, mineral supplements, rainwater

## Abstract

Herein, we developed a colorimetric method for the determination of Cu^2+^ and Zn^2+^ using NBD-G as a novel selective metal sensor. NBD-G was easily synthesized by a nucleophilic substitution reaction between 4-chloro-7-nitro-2,1,3-benzoxadiazole (NBD-Cl) and Girard’s Reagent P. The NBD-G solution is yellow, but when it reacts with Cu^2+^ and Zn^2+^, its color changes selectively to red (510 nm) and orange (480 nm), respectively. NBD-G was used as a sensor for Cu^2+^ and Zn^2+^, showing a high sensitivity down to 0.77 µM for Cu^2+^ and 1.66 µM for Zn^2+^. NBD-G could determine both metals simultaneously; thus, it was applied to determine them in multimineral supplements, which showed excellent recoveries. Next, a filter paper impregnated with NBD-G was prepared as a test paper, and a simple, selective, and rapid onsite method for quantifying Cu^2+^ was developed as, interestingly, the paper showed no change upon the addition of Zn^2+^. Next, Cu^2+^ could be quantified with high selectivity and accuracy by photographing the color change with a smartphone camera and processing the image with Image J. The detection limit for Cu^2+^ using this method was 3.9 µM. Finally, the NBD-G test paper method was able to satisfactorily quantify Cu^2+^ spiked into the rainwater.

## 1. Introduction

Heavy metals are one of the toxic substances that adversely affect human health, and environmental pollution by heavy metals has long been an issue of concern [1,2,3]. In general, heavy metals are not biodegradable, and therefore they tend to persist in the environment and cause various toxic effects on living organisms [4,5]. For example, excessive intake of copper can cause diseases such as Wilson’s disease and Parkinson’s disease [6,7]. Zinc is a metal that plays a major role in biological functions such as immune function and enzyme activity, but its deficiency or excessive intake is associated with diseases such as brain damage and Alzheimer’s disease [8,9]. Heavy metals present in the environment, even in trace amounts, can be concentrated throughout the food chain, resulting in the accumulation of heavy metals, which can cause serious health problems [10]. In the case of a health problem in which heavy metals such as copper and zinc are suspected to be involved, it is important to rapidly monitor the presence of heavy metals in the environment onsite to determine their contamination status [11]. Therefore, it is necessary to develop a simple, rapid, and selective method to measure heavy metals.

Currently, atomic absorption spectrometry is widely used as a method for measuring heavy metals [12,13]. Atomic absorption spectrometry is based on the ability of each metal element to absorb light at a specific wavelength and is capable of highly sensitive and selective measurements. However, it is not easy to perform onsite analysis in areas where heavy metal contamination is suspected because of the relatively large equipment and complicated measurement procedures. On the other hand, the measurement method using chromogenic or fluorescent reagents, which change color by reacting with heavy metals, is simple to operate and can be visually detected, making it a suitable method for onsite analysis [14,15].

Many coloring and fluorescent reagents have been developed for the measurement of copper and zinc ions in environmental and biological samples [16,17]. Cu^2+^ typically induces static fluorescence quenching of heterocyclic nitrogenous rings [18,19], whereas Zn^2+^ causes fluorescence enhancement in various organic species [20]. However, most of these reagents are applicable to only one metal ion [21], and when multiple metal ions are measured, it is necessary to use different reagents, which complicates the measurement operation. One solution to this problem is to use multifunctional reagents that show different responses depending on the type of metal ion; however, there are few examples of reagents that show different color changes depending on the type of metal ion, and their synthetic reactions are complex [22,23]. In addition, the multifunctional reagents that have been developed thus far have the disadvantage that they do not respond under the same measurement conditions [24] and that the signal intensity differs greatly depending on the target of measurement, resulting in a large difference in sensitivity [25].

Some reports have mentioned the reactivity of aromatic compounds substituted with hydrazide groups towards Zn^2+^ [26] and Cu^2+^ [27,28,29]. This encouraged us to test a library of aromatic hydrazide derivatives by reacting 4-chloro-7-nitro-2,1,3-benzoxadiazole (NBD-Cl) with different hydrazide compounds and testing their responses to Zn^2+^ and Cu^2+^. Among the tested compounds, NBD-G, which is the result of the reaction between NBD-Cl and 1-(hydrazinocarbonylmethyl)pyridinium chloride (Girard’s Reagent P), showed a colorimetric response to Zn^2+^ and Cu^2+^ by changing its color from yellow to orange and red, respectively. Hence, NBD-G was used as a new chemosensor for Zn^2+^ and Cu^2+^ detection.

Smartphone-integrated assays [30,31], especially paper-based ones [32,33], have many advantages in terms of an onsite and rapid assay of target analytes using very cheap utilities and instruments [34]. Hence, a filter paper impregnated with NBD-G was prepared as a test paper, and a simple and rapid onsite method for quantifying Cu^2+^ was developed, as the prepared NBD-G test paper instantly turned red when a Cu^2+^ solution was added while showing no change upon the addition of Zn^2+^. Moreover, Cu^2+^ can be quantified in environmental water samples with high selectivity and accuracy by photographing the color change with a smartphone camera and processing the image with ImageJ.

## 2. Materials and Methods

### 2.1. Materials and Instrumentation

The NBD-Cl was purchased from Sigma-Aldrich (St. Louis, MO, USA). Formohydrazide (FH), acetohydrazide (AH), benzoylhydrazine (BH), Girard’s Reagent P (1-(hydrazino-carbonyl methyl)pyridinium chloride, GRP), terephthalic dihydrazide (TH), 3-hydroxy-2-naphthoic acid hydrazide (HNH), and 2-(4-hydrazinocarbonylphenyl)-4,5-diphenylimidazole (HCPI), 2,4-dinitrophenylhydrazine (DNPH), hydrazine (HZ), and phenylhydrazine (PH) were from Tokyo Chemical Industries (Tokyo, Japan). Different salts, including silver nitrate (Ag(NO)_3_), aluminum nitrate hexahydrate (Al(NO_3_)_3_·6H_2_O), barium chloride (BaCl2), cobalt chloride hexahydrate(CoCl_2_·6H_2_O), ferrous chloride tetrahydrate (FeCl_2_·4H_2_O), ferric chloride hexahydrate (FeCl_3_·6H_2_O), potassium chloride (KCl), nickel acetate tetrahydrate (Ni(CH_3_COO)·4H_2_O, zinc acetate bihydrate (Zn(CH_3_COO)_2_·2H_2_O), and strontium chloride (SrCl_2_) were obtained from Wako Pure Chemicals (Osaka, Japan). Sodium chloride (NaCl) and calcium chloride (CaCl_2_) were purchased from Nacalai Tesque (Kyoto, Japan). Copper acetate dihydrate (Cu(CH_3_COO)_2_·H_2_O), magnesium chloride (MgCl_2_), and manganese chloride (MnCl_2_) were purchased from Kishida Chemical Co. Ltd. (Osaka). The water used throughout the experiments was purified distilled water obtained by passing distilled water through a Yamato Autosill WG220 pure water generator from Yamato Scientific Co., Ltd. (Tokyo, Japan).

Spectrophotometric measurements and UV–visible spectra were recorded using a Shimadzu UV-1800 spectrophotometer (Kyoto, Japan). In the fast screening of different chemosensors, a 96-well Microplate SPL Life Sciences 96-well PS White was used and photographed using a mobile camera. To measure the molecular ion peaks and record the mass spectra, a Quattro micro™ triple-quadrupole mass spectrometer equipped with an electrospray ionization (ESI) source (Waters Co., Milford, MA, USA) and a JEOL JMS-DX 303 electron impact mass spectrometer (Jeol Ltd., Tokyo, Japan) were used. For the elemental analysis of NBD-G, a PerkinElmer 2400 Series II elemental analyzer (Waltham, MA, USA) was used. The ^1^H-NMR spectrum of NBD-G in DMSO-d6 was measured using a Varian 5-inova 500 (500 Hz) spectrometer from Agilent Technologies, Inc. (Santa Clara, CA, USA).

### 2.2. Procedure for the Screening of NBD Derivatives as Metal Sensors

First, we screened new reagents for metal measurements using the NBD-Cl reaction products with hydrazide and hydrazine reagents. A 0.2 mM of NBD-Cl and 0.2 mM of each hydrazide or hydrazine reagent were mixed in equal amounts in MeOH in a test tube and heated at 80 °C for four hours to synthesize condensed compounds of these reagents. Then, to 100 μL of reagent solution, 100 μL of aqueous metal ion solutions (Na^+^, K^+^, Co^2+^, Cu^2+^, Mg^2+^, Mn^2+^, Ni^2+^, Sn^2+^, Sr^2+^, Zn^2+^, Fe^3+^) with concentrations of 0.2 mM were added to each well without purification, and changes in fluorescence and color tone were observed.

### 2.3. Synthesis of NBD-G

**Scheme 1:**
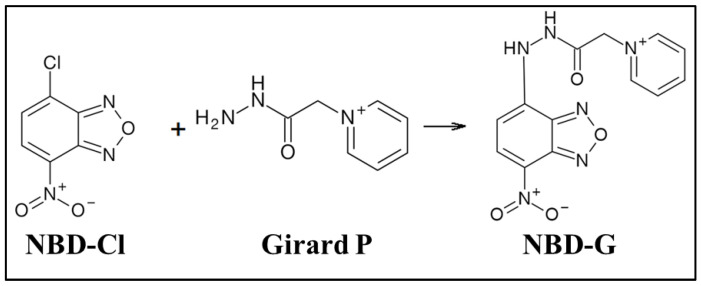
The reaction used for the synthesis of NBD-G from NBD-Cl and Girard’s P Reagents.

The procedure for the synthesis of NBD-G is shown in Scheme 1. NBD-Cl (200 mg, one mmol) was mixed with Girard’s Reagent P (188 mg, one mmol) in 6 mL of 1,4-dioxane, and 3 mL of MeOH and sonicated for 10 min. Then, 200 µL of 125 mM borate buffer (pH 10.0) was added to adjust the pH of the solution to basicity, and crude crystals were obtained by heating at 50 °C for 2.5 h. The crude crystals were filtered by suction and recrystallized in MeOH to obtain yellow crystals of NBD-G (93 mg, 26.6%).

**Figure 1:**
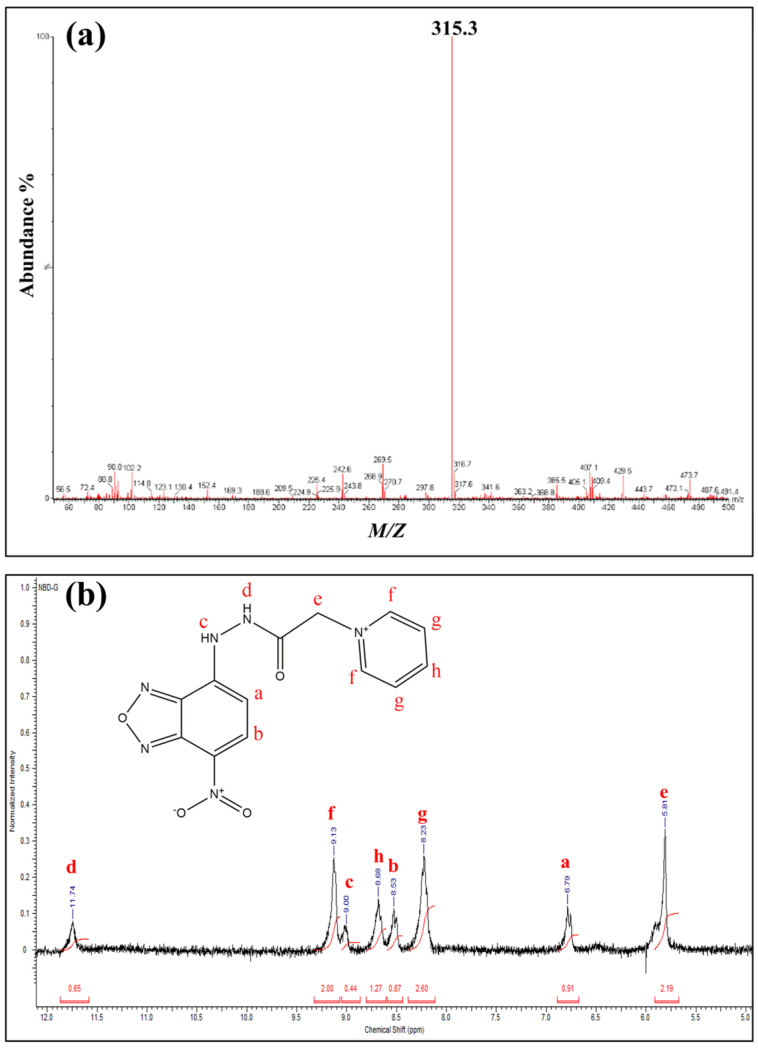
Characterization of NBD-G, where (**a**) is the ESI-MS spectrum of NBD-G showing the molecular ion peak at *m*/*z* of 315.3, and (**b**) is the ^1^H-NMR spectrum of NBD-G.

The resulting crystals were subjected to elemental analysis and ESI-MS to confirm their structures. As a result, a molecular ion peak corresponding to *m*/*z* = 315 (Figure 1a), the molecular weight of NBD-G, was detected. The results of elemental analysis of the synthesized compound were C, 44.46%; H, 2.88%; N, 23.87% (calculated for C_13_H_11_N_6_O_4_: C,44.52%; H, 3.16%; N, 23.96%), which are in good agreement with theoretical values. Furthermore, the NBD-G ^1^H-NMR spectrum was measured (Figure 1b) and exhibited the subsequent chemical shifts were found (δ, ppm): δ 5.81 (2 H, CH_2_, s), δ 6.79 (1 H, Benzofuran-H, d), δ 8.23 (2 H, Pyridine-H, t), δ 8.53 (1 H, Benzofuran-H, d), δ 8.68 (1 H, Pyridine-H, t), δ 11.5 (1 H, NH, S), δ 9.13 (2 H, Pyridine-H, s), and δ 11.5 (1 H, NH, S). The results of ESI-MS, ^1^H-NMR, and elemental analysis confirmed that the targeted sensor, NBD-G, was synthesized with a high purity.

### 2.4. Measurement of Cu^2+^ and Zn^2+^ Using NBD-G as a Chemosensor

To 1.0 mL of 50.0 μM NBD-G, 1.0 mL aqueous solutions of different concentrations of Cu^2+^, Zn^2+^, or both were added. Then, the UV–visible spectra were recorded, and the absorbance was measured at 480 nm and 540 nm for Zn^2+^ and Cu^2+^, respectively. A blank experiment using water instead of metals was performed at the same time. A calibration curve was plotted for each Zn and Cu in the range of 2.5 to 50.0 μM by plotting the concentration of the metals vs. the corresponding absorbance at 480 nm and 540 nm for Zn^2+^ and Cu^2+^, respectively.

### 2.5. Measurement of Cu^2+^ by NBD-G Impregnated Test Paper

NBD-G test paper was prepared by soaking cellulose filter paper (ADVANTEC, Paper disc) in 200 µM NBD-G solution for 15 min and then lifted to dry at room temperature. Next, 20 μL of the Cu^2+^ solution was dropped onto the NBD-G test paper. The color change in the test paper was photographed using a mobile phone camera. The photos were taken on the normal room light by a phone fixed 30 cm perpendicular to the test papers one minute after the addition of Cu^2+^ solution to the NBD-G test paper and analyzed using Image J free software ver. 1.54k. The green color intensity was extracted from the photographs using ImageJ and was used as an indicator of the change in the test paper from yellow to red. The signal intensity of Green (I_sample_) at an arbitrary area in the image of the test paper after Cu^2+^ addition and the signal intensity of Green (I_blank_) in the image of the test paper with water added as a blank were extracted using Image J software, and the relative signal intensity Y was calculated according to previous reports [35,36] as follows:

$$Y=1000\times \left(1-\frac{\mathrm{Isample}}{\mathrm{Iblank}}\right)$$

The same procedure was followed for the analysis of Cu^2+^ in environmental water where 20 μL of environmental water samples spiked with different concentrations of Cu^2+^ was added to the test papers, and the same previous procedure was followed.

## 3. Results

### 3.1. Screening of NBD Derivatives as Metal Sensors

**Figure 2:**
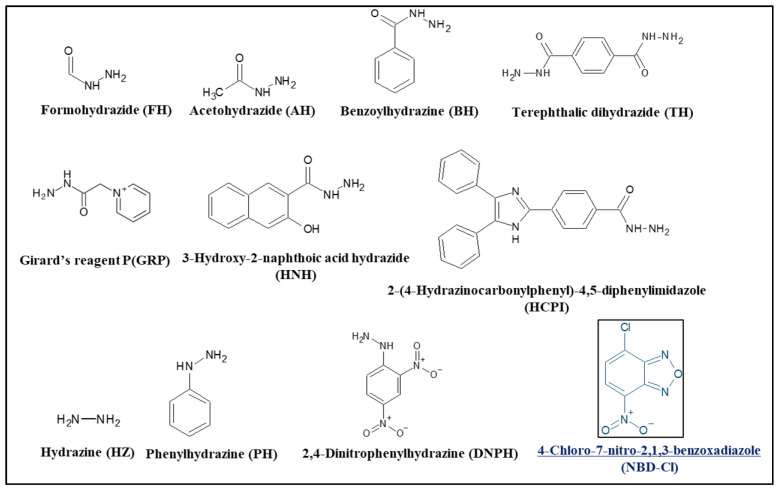
Structure of the tested hydrazide and hydrazine derivative substrates and NBD-Cl.

As synthetic raw materials for metal measuring reagents, NBD-Cl was reacted with seven types of hydrazides: formohydrazide (FH), acetohydrazide (AH), benzoylhydrazine (BH), Girard’s Reagent P (GRP), terephthalic dihydrazide (TH), 3-hydroxy-2-naphthoic acid hydrazide (HNH), and 2-(4-hydrazinocarbonylphenyl)-4,5-diphenylimidazole (HCPI) and three types of hydrazine: 2,4-dinitrophenylhydrazine (DNPH), hydrazine (HZ), and phenylhydrazine (PH) (Figure 2). All the tested hydrazine and hydrazide were commercially available except for HCPI, which was synthesized as previously reported by our research group [37]. The synthesis was carried out using equimolar solutions of NBD-Cl, hydrazide, and hydrazine derivatives. Then, the formed products without purification were reacted with equimolar concentrations of different metal ion solutions (Na^+^, K^+^, Co^2+^, Cu^2+^, Mg^2+^, Mn^2+^, Ni^2+^, Sn^2+^, Sr^2+^, Zn^2+^, Fe^3+^), and changes in fluorescence and color tone were observed.

Contrary to our initial expectation, fluorescence was not observed in the reaction solutions of NBD-Cl with hydrazines and hydrazides, even when any metal ion was added. Therefore, we evaluated the utility of the synthesized compounds as colorimetric sensors.

**Figure 3:**
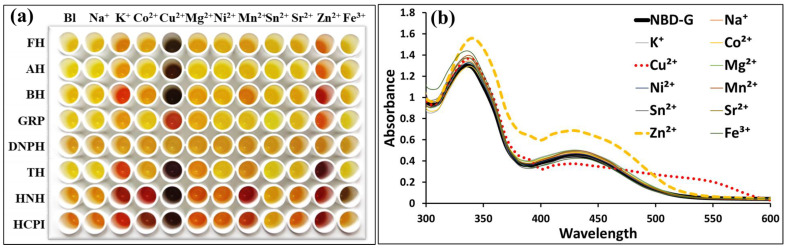
Screening of NBD-G among different proposed colorimetric sensors (**a**) showing the color response of reaction products of NBD-Cl with various hydrazides or hydrazines towards different metal ions, and (**b**) the absorbance spectra of NBD-g in the absence and in the presence of different metal ions, where these spectra were recorded without subtraction of reagent blank.

A photograph of the 96-well microplate immediately after the addition of metal ions to each well containing the reaction product of NBD-Cl with different hydrazides and hydrazines is shown in Figure 3. The leftmost column is blank (Bl) with purified water without metal ions. By comparing the color tones of this column with those of the columns to which metal ions were added, we evaluated the coloration caused by the reaction between the metal ions and the compounds formed in the wells. For the compounds formed by the reaction of NBD-Cl with hydrazides, the color tone was changed by the addition of Cu^2+^ and Zn^2+^, suggesting that these compounds can be used for the colorimetric measurement of Cu^2+^ or Zn^2+^. In contrast, no color change was observed in the reactions of NBD-Cl and DNPH. Therefore, it is possible that the hydrazide structure is important for the coloration reaction of Cu^2+^ and Zn^2+^. The reaction products of BH and TH responded to K^+^, and the reaction products of HNH showed a relatively intense color change in response to Mn^2+^ and a less notable color change to K^+^, Co^2+^, Ni^2+^, Fe^3+^, and Mg^2+^. The degree of coloration of these products is weak, and they are not considered suitable reagents for measurement because of the large number of competing metal ions. Among the compounds that were found to be useful as reagents for the colorimetric measurement of Cu^2+^ or Zn^2+^, black precipitates were observed after the reaction, except for the reaction products of NBD-Cl and GRP (Figure 3, NBD-G). The coloration with metal ions was also most pronounced for NBD-G, suggesting that NBD-G is the most suitable reagent for the measurement of Cu^2+^ or Zn^2+^. This was confirmed by measuring the absorbance spectra of the NBD-G alone and in the presence of different metal ions. As shown in Figure 3b, only Cu^2+^ and Zn^2+^ caused significant changes in the spectrum of NBD-G, which demonstrates the high selectivity of the probe for these two metals. Regarding other tested NBD derivatives, their absorbance spectra alone and in the presence of different metal ions are illustrated in Figure S1 in the Supplementary Materials.

**Figure 4:**
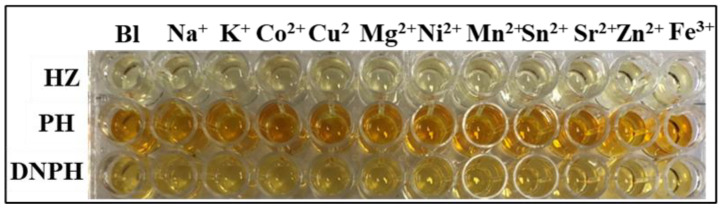
Color response of reaction products of NBD-Cl with various hydrazine towards different metal ions.

As shown in Figure 3, unlike the reaction products of hydrazides, the reaction products of NBD-Cl and DNPH did not change their color tone when reacted with Cu^2+^ and Zn^2+^, suggesting that the hydrazide structure may be important in the color reaction with these metal ions. In order to confirm this effect, we observed the color change in the solutions of three hydrazines, including DNPH, reacted with NBD-Cl by adding metal ions, including Cu^2+^, in the same manner. As shown in Figure 4, the reaction products of hydrazine and NBD-Cl did not exhibit any color change for any of the 11 metal ions. Therefore, it is considered that the hydrazide structure is necessary for color change due to the reaction with metal ions.

### 3.2. Selective Response of NBD-G to Cu^2+^ and Zn^2+^

From our initial screening of the different NBD–hydrazide reagents discussed in the previous section, NBD-G was the sensor of choice for the determination of Cu^2+^ and Zn^2+^. To thoroughly evaluate the selectivity of NBD-G for Cu^2+^ and Zn^2+^, the changes in the absorbance of 15 metal ions (Ag^+^, Al^3+^, Ba^2+^, Ca^2+^, Cu^2+^, Co^2+^, Fe^2+^, Fe^3+^, K^+^, Mg^2+^, Mn^2+^, Na^+^, Ni^2+^, Sr^2+^, and Zn^2+^) were mixed with purified NBD-G. Changes in the absorbance of solutions of NBD-G mixed with purified NBD-G were investigated. Aqueous solutions of each metal ion were added dropwise to a MeOH solution of NBD-G, and the absorption spectra of the reaction solutions were measured after 1 min of incubation.

**Figure 5:**
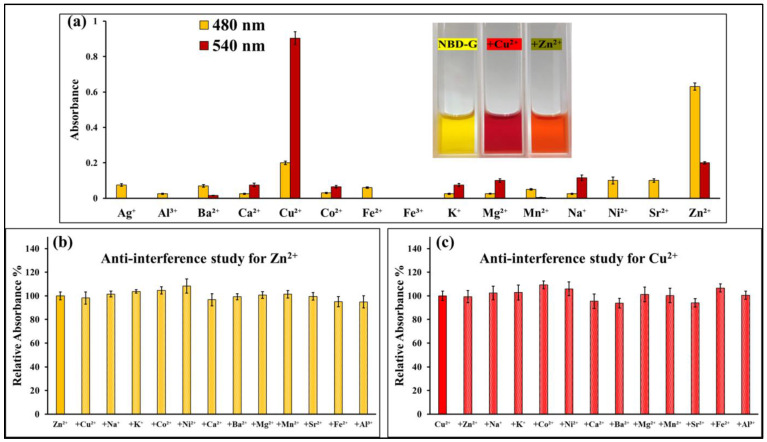
Selectivity and anti-interference study. (**a**) The response of NBD-G towards different metal ions at 480 and 540 nm. Only Cu^2+^ and Zn^2+^ showed significant response at 540 and 480 nm, respectively. The metals that showed no columns indicate that they have no absorbance at the specified wavelengths. The inset shows the color change in NBD-G (100 µM) in MeOH after the addition of a few drops of Cu^2+^ (10.0 mM) and Zn^2+^ (10.0 mM). (**b**) shows the response of NBD-G + Zn^2+^ alone and in the presence of different metal ions (five equivalents), and (**c**) shows the response of NBD-G + Cu^2+^ alone and in the presence of different metal ions (five equivalents).

Figure 5 shows the difference in absorbance at wavelengths of 480 nm and 540 nm before and after the reaction with metal ions. The absorbance of NBD-G increased at 480 nm due to the reaction with Zn^2+^ and at 540 nm due to the reaction with Cu^2+^. However, NBD-G did not show significant changes in the absorbance for the other metal ions. These results confirm the results obtained in the previous section that NBD-G is useful for selective colorimetric detection of Cu^2+^ and Zn^2+^. Furthermore, since the absorbance changes at different wavelengths depending on the reaction with Cu^2+^ and Zn^2+^, it is considered that the concentration of Cu^2+^ and Zn^2+^ in the same sample can be separately quantified.

The color tone of NBD-G in the MeOH solution was changed by adding drops of 1.0 mM Cu^2+^ and Zn^2+^ to the solution. The MeOH solution of NBD-G itself showed a yellowish color, and when Cu^2+^ and Zn^2+^ solutions were added dropwise, NBD-G instantly showed a red and orange coloration, respectively. Therefore, it is considered that NBD-G can be used for the visual detection of Cu^2+^ and Zn^2+^ (Figure 5 inset). Additionally, a competitive anti-interference study was conducted by recording the absorbance of NBD-G complexes with Cu^2+^ and Zn^2+^ in the presence of different competing metals, and the results are shown in Figure 5b,c. It was found that Cu and Zn could be detected with very high anti-interference ability of NBD-G.

### 3.3. Quantification of Cu^2+^ and Zn^2+^ with NBD-G

**Figure 6:**
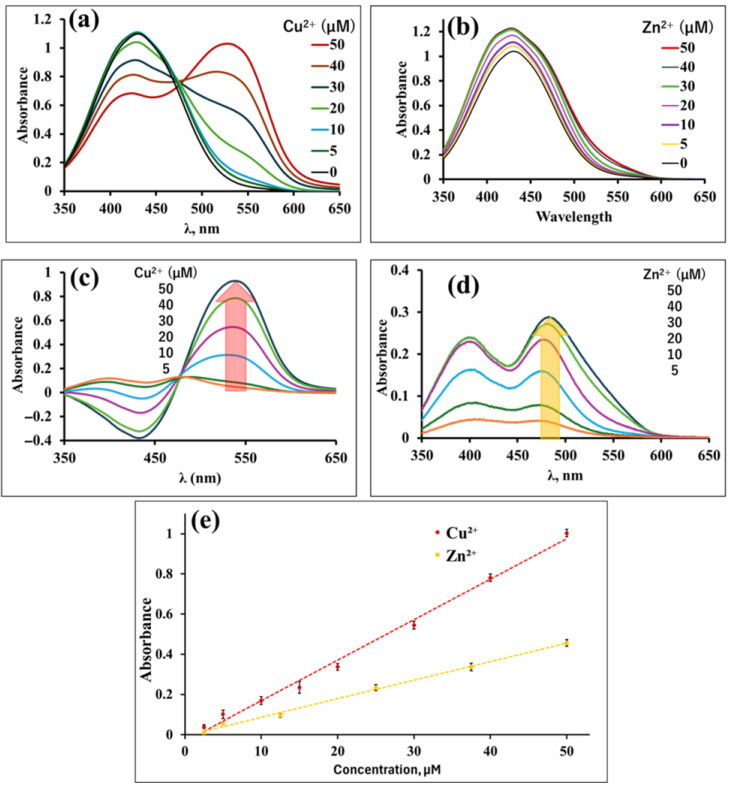
Summary of the quantitative response of NBD-G towards Cu^2+^ and Zn^2+^ showing the spectral change in NBD-G (50 µM) upon reaction with (**a**) Cu^2+^ (0–50 µM) and (**b**) Zn^2+^ (0–50 µM) recorded vs. the solvents as blank. Figure (**c**,**d**) show the absorbance spectra of NBD-G complexes with (**c**) Cu^2+^ (0–50 µM) and (**d**) Zn^2+^ (0–50 µM) recorded against NBD-G as blank, while (**e**) shows the calibration curve for detecting Cu^2+^ (2.5–50 µM) and Zn^2+^ (2.5–50 µM) using NBD-G (50 µM).

At first, the concentration of NBD-G for sensing Cu^2+^ and Zn^2+^ was optimized in a preliminary study. Several concentrations of NBD-G were tested while using 50 μm Cu^2+^ and Zn^2+^. The best absorbance was obtained using 50 μM NBD-G for both metals. Then, the changes in the absorption spectrum of NBD-G after the addition of various concentrations of Cu^2+^ were observed. Equal amounts of 50 µM NBD-G in MeOH and different concentrations of Cu(CH_3_COO)_2_ (0–50 µM) were mixed, and the absorption spectrum was measured after 1 min using a UV–visible absorption spectrophotometer. Figure 6a,c show the changes in the NBD-G spectra one minute after the addition of different concentrations of Cu(CH_3_COO)_2_ using solvents (Figure 6a) and NBD-G (Figure 6c) as blank. The MeOH solution of NBD-G was a yellow solution with an absorption maximum near 430 nm, but the addition of Cu^2+^ caused a spectral change with a decrease in absorbance near 430 nm and an increase in absorbance near 540 nm, with 480 nm as the isosbestic point, and the solution turned red.

Similarly, the change in the absorption spectrum of NBD-G owing to the addition of Zn^2+^ was studied using the solvent and NBD-G as blank, and the results are shown in Figure 6b,d, respectively. The addition of Zn^2+^ to the NBD-G solution increased the absorbance at the maximum absorption wavelength of 480 nm (Figure 6d), and the color of the solution changed from yellow to orange. The reaction between NBD-G and Zn^2+^ was different from that with Cu^2+^, where there was no wavelength region in which the absorbance decreased, and no isosbestic point was observed.

A colorimetric method for the determination of Cu^2+^ and Zn^2+^ using NBD-G as a reagent was developed. An amount of 1.0 mL of a MeOH solution of 50 µM NBD-G and 1.0 mL of the solution of Cu^2+^ or Zn^2+^ were mixed and allowed to stand for 1 min, and then the absorbance of Cu^2+^ and Zn^2+^ was measured at wavelengths of 540 nm and 480 nm, respectively, versus NBD-G as blank. A calibration curve was constructed using the standard solution of Cu^2+^, and high linearity with a correlation coefficient of 0.997 was obtained in the concentration range of 2.5–50 µM (Figure 6c, Table 1). Similarly, a calibration curve was constructed using a standard solution of Zn^2+^, and a correlation coefficient of 0.998 was obtained in the concentration range of 2.5–50 µM. When the lower detection limit is defined as blank+3SD [18,38], the lower detection limits for Cu^2+^ and Zn^2+^ are calculated to be 0.77 µM and 1.66 µM, respectively. These detection limits are lower than the water quality standards based on the Water Supply Law defined by the Ministry of Health, Labour and Welfare of Japan, and NBD-G is considered to be useful for monitoring Cu^2+^ and Zn^2+^ in water samples.

**Figure 7:**
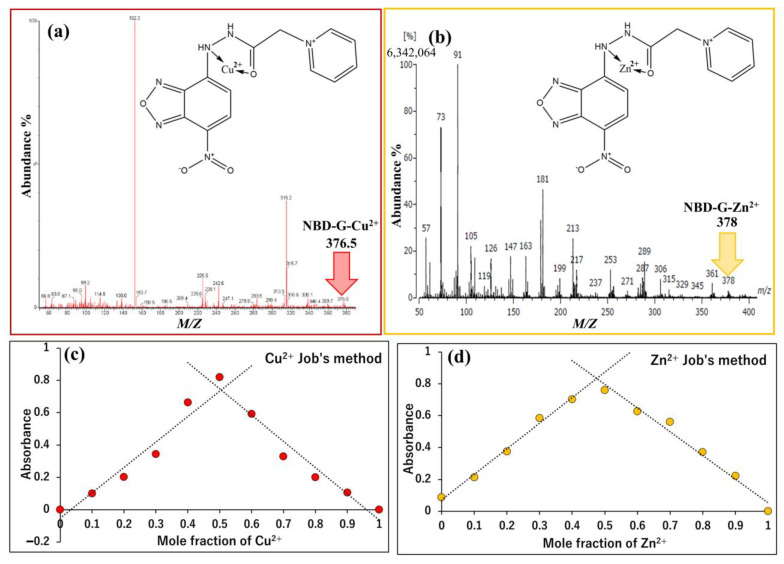
Characterization of NBD-G complexes, presenting the mass spectra of the complex of NBD-G with (**a**) Cu^2+^ and (**b**) Zn^2+^, and Job’s plot of continuous variation for (**c**) Cu^2+^ and (**d**) Zn^2+^. The insets in (**a**,**b**) show the possible structure of the complexes.

Next, the complexation of NBD-G with Cu^2+^ and Zn^2+^ was studied by mass spectrometry. The mass spectrum of the NBD-G complex with Cu^2+^ and Zn^2+^ showed molecular ion peaks at 376.5 and 378, respectively (Figure 7a,b), corresponding to their 1:1 complex composition. The possible structures of the complexes are shown in the insets in Figure 7a,b, where the metals bind coordinately with the terminal N and the carbonyl O of the hydrazide group [39,40,41]. Furthermore, Job’s method of continuous variation was adopted to confirm the molar ratio of the metals to NBD-G in the formed complex. As shown in Figure 7c,d, the ratio is 1:1 for the complexes of the two metal ions with NBD-G.

### 3.4. Quantification of Cu^2+^ and Zn^2+^ in the Presence of Each Other and Application to Multimineral Supplements

As Cu and Zn are usually present together in mineral supplements, the determination of each one of them in the presence of 10 μM of the other was tested. The data are summarized in Table 2. The found % of Zn^2+^ in the presence of 10 μM Cu^2+^ was acceptable, ranging from 102% to 112.3%, whereas that of Cu^2+^ in the presence of 10 μM Zn^2+^ was excellent, ranging from 95.0% to 104.4%, demonstrating the ability of the proposed colorimetric method to determine both metals in the presence of each other. This was attributed to the fact that the NBD-G-Zn^2+^ complex was measured at the isosbestic point (480 nm) of the NBD-G-Cu^2+^ complex. NBD-G-Cu^2+^ was measured at 540 nm, and NBD-G-Zn^2+^ exhibited weak absorbance.

The previous results encouraged us to determine Cu^2+^ and Zn^2+^ in the combined multimineral supplement available in drug stores in Japan under the name Moringa multimineral jelly drink. This supplement is labeled to contain 227 mg calcium, 2.3 mg iron, 35 mg magnesium, 0.3 mg copper, and 2.9 mg zinc metal ions. The nominal content of Cu^2+^ and Zn^2+^ in this supplement was determined using the proposed colorimetric sensor and was found to be 0.29 mg and 2.7 mg for Cu^2+^ and Zn^2+^, respectively. The obtained recoveries % was 96.7% for Cu^2+^ and 93.1% for Zn^2+^, demonstrating the excellent applicability of the proposed method for simultaneous determination of Cu^2+^ and Zn^2+^ in a complicated matrix that contains multi-minerals.

### 3.5. Application to NBD-G Test Paper for the Determination of Cu^2+^

**Figure 8:**
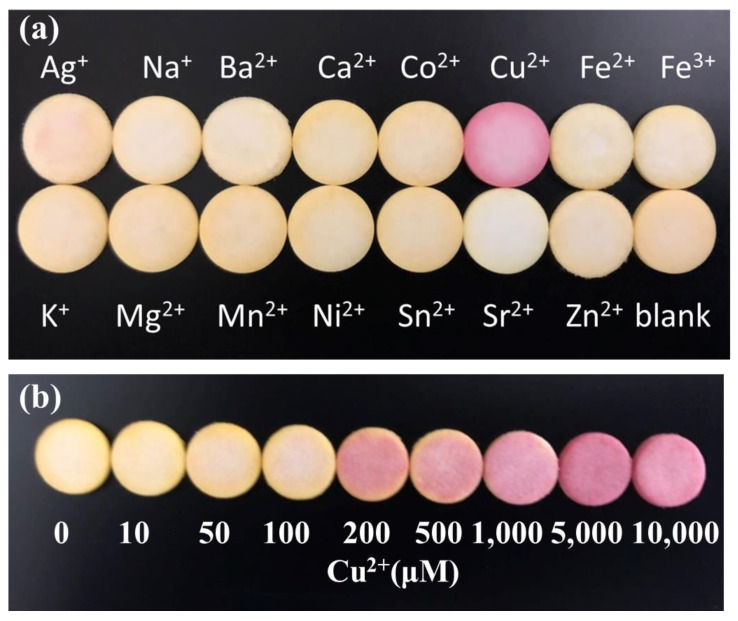
Selectivity study of the test paper method, showing the response of NBD-G-impregnated test paper (200.0 μM NBD-G) to (**a**) different metal ions and (**b**) different concentrations of Cu^2+^.

The reaction of NBD-G with Cu^2+^ is rapid, and the color of the solution instantly changes from yellow to red. Therefore, we attempted to apply NBD-G as a test paper method for the simple and rapid detection of Cu^2+^ by taking advantage of this property. The NBD-G test paper was prepared as mentioned in the experimental section, and then different metal ions were dropped into it. Afterward, the filter papers were photographed, and the results are shown in Figure 8. Only Cu^2+^ changed the color of the test paper from yellow to red, whereas other metals, including Zn, did not cause a significant change in the color of the test paper. The reason for the unexpected non-reactivity of the test paper to Zn is unknown; however, it could be due to the nature of the reaction between NBD-G and Zn, which necessitates a solid-state medium.

The results in Figure 8 clearly demonstrate the selectivity of the test paper to Cu^2+^, which encouraged us to use the test paper coupled with a mobile camera for the quantitative determination of Cu^2+^. Different concentrations of Cu^2+^ were dropped on the NBD-G test paper, and, consequently, the color of the test paper changed from yellow to red, as shown in Figure 8b, where the higher the Cu^2+^ concentration, the stronger the color change observed. Based on this result, it is considered that the NBD-G test paper can be used for the simple and rapid semi-quantitative determination of Cu^2+^.

To develop the NBD-G test paper into a simple onsite determination method for Cu^2+^, we attempted to develop an image processing method by capturing the color tone changes in the test paper using the camera function of a smartphone. In this study, ImageJ, a software program that can quantify the intensity of red, green, and blue signals in a specified area of an image, was used for image analysis. This is expected to improve the quantification of the NBD-G test paper, which can only be semi-quantified by visual inspection. Furthermore, since ImageJ can be used as a smartphone application, it is considered to be a method suitable for onsite quantitative analysis that does not require a dedicated measuring device or computer.

**Figure 9:**
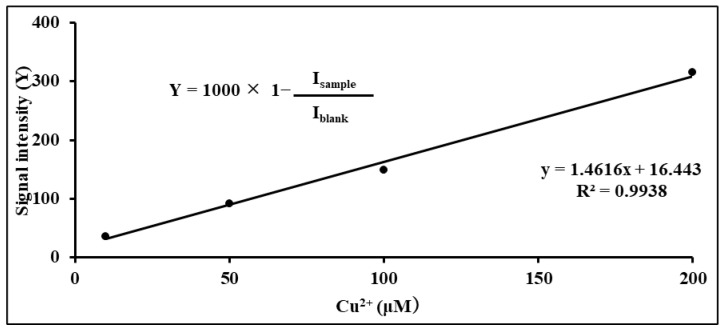
Calibration curve of Cu^2+^ using the proposed test paper method impregnated with 200 μM NBD-G.

As a result of the investigation of the image analysis processing method for the simple quantification method using NBD-G test papers, a calibration curve of the Cu^2+^ standard solution was generated, as discussed previously in the experimental section. The images were taken by a smartphone camera one minute after the addition of Cu^2+^ solution to the NBD-G test paper. The green color intensity was obtained by analyzing the images of the test papers using ImageJ, which was used as an indicator of the change in the test paper from yellow to red, as mentioned in the experimental section. Image J software can extract the intensity of the red, blue, and green colors intensities. Only the green color intensity was decreased quantitatively. That is because the yellow color is known to be formed from an equal intensity of red and green colors, and when the yellow color turns to red, this happens through the decrease in the green color intensity. The plot of the relative signal intensity Y versus the concentration of Cu^2+^ solution added to the samples showed good linearity with a correlation coefficient of 0.998 in the range of 10–200 µM, with an LOD of 3.9 µM (Figure 9), confirming that this method has sufficient quantitative performance. The detection limit of this method, calculated according to the ICH guidelines [42] by dividing the standard deviation of one of the residuals (10.0 µM Cu^2+^ solution) by the slope of the calibration curve, was 3.9 µM, indicating that the sensitivity of this method is comparable to that of the method using a UV–visible spectrophotometer.

The sensitivity of the NBD-G assay was compared with that of previously reported assays for Cu^2+^ using other chromogenic reagents (Table 3). The sensitivity of the NBD-G method is 6.5–8.5 times higher than that of other previously reported methods [43,44,45,46].

The accuracy and repeatability of the test paper method were evaluated at four concentrations of Cu^2+^ within the calibration curve range. The test papers were prepared three times in the same way as the calibration curve was prepared, and the color changes in the test papers to which each concentration of Cu^2+^ solution was added were converted into Cu^2+^ concentrations by image analysis (Table 4). As a result, the accuracy (found %) ranged from 85.2 to 110.6%, and the reproducibility (*n* = 3) was excellent with RSD ≤ 5.2%.

### 3.6. Application of NBD-G Test Paper for Environmental Analysis of Cu^2+^

**Figure 10:**
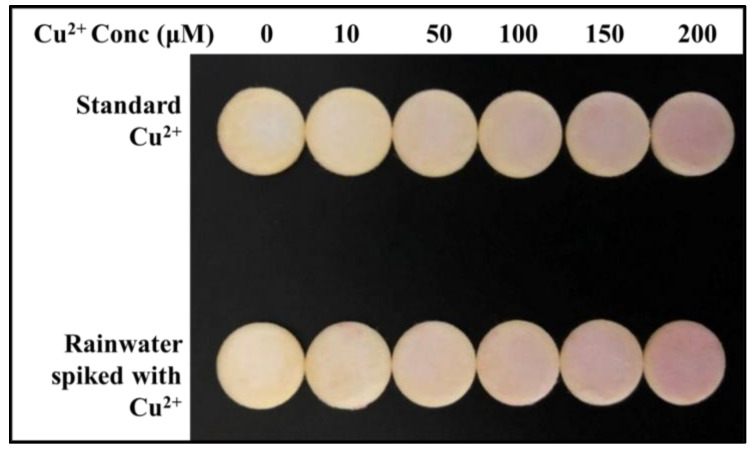
Color change in NBD-G impregnated test paper upon addition of rainwater (**lower** line) and pure water (**upper** line) spiked with Cu^2+^.

In order to evaluate the practicality of the NBD-G test paper method, the determination of Cu^2+^ in rainwater was conducted. After the filtration of rainwater collected in Nagasaki City, samples of 10–200 µM Cu^2+^ were dropped onto NBD-G test paper and photographed using a smartphone for quantification by image processing (Figure 10, Table 5). The recovery rate was calculated by comparing the results with those of Cu^2+^ standard solutions of the same concentration, and the results were 99.3–106.2%, which is excellent, indicating that the Cu^2+^ concentration in environmental water can be determined using this test paper method. The NBD-G test paper method enables the simple determination of Cu^2+^ based on the results obtained using a smartphone camera, and its sensitivity is comparable to that of the instrumental analysis method, making it a powerful tool for the onsite analysis of Cu^2+^ in the environment.

## 4. Conclusions

In this study, we developed a chromogenic determination method for Cu^2+^ and Zn^2+^ using NBD-G, which was found to be useful as a reagent for metal determination in a pre-screening study. The NBD-G solution was yellow in color but changed to red and orange when reacted with Cu^2+^ and Zn^2+^, respectively. The absorption spectra of the NBD-G solution before and after the reaction with Cu^2+^ and Zn^2+^ were measured, and the results showed that the absorbance at 480 nm increased after the reaction with Zn^2+^ and the absorbance at 540 nm increased after the reaction with Cu^2+^. The color tone and absorption spectrum of NBD-G did not change with the addition of other metal ions, suggesting that NBD-G reacts selectively with Cu^2+^ and Zn^2+^. Therefore, we developed an absorption spectrophotometric method for the determination of Cu^2+^ and Zn^2+^ using NBD-G as the reagent. As a result, it was confirmed that Cu^2+^ and Zn^2+^ could be determined by measuring the absorbance at wavelengths of 540 nm and 480 nm, and the lower detection limits were 0.77 µM for Cu^2+^ and 1.66 µM for Zn^2+^. Furthermore, it was found that the simultaneous determination of Cu^2+^ and Zn^2+^ in the presence of each other, either in synthetic mixtures or in a multi-mineral supplement, is possible by selecting the appropriate wavelength for each of them.

Next, we developed a simple and rapid onsite selective method for the determination of Cu^2+^ using NBD-G-impregnated filter paper as a test paper. The prepared NBD-G test paper instantly turned red upon the addition of the Cu^2+^ solution, and the color change was captured by a smartphone camera and processed by image processing, indicating that Cu^2+^ can be determined with high simplicity. The detection limit of Cu^2+^ using this method is 3.9 µM, which is comparable to the sensitivity of the absorption spectrophotometric method using a UV–visible spectrophotometer. Finally, the NBD-G test paper method could quantify Cu^2+^ in environmental water samples.

## Figures and Tables

**Table 1:** Calibration curve for Cu^2+^ and Zn^2+^.

Metal (λmax)	Range, µM	Equation ^1^	r	LOD ^2^, µM
Cu^2+^ (540 nm)	2.5–50	Y = 2.00 × 10^−2^ X − 3.3 × 10^−2^	0.997	0.77
Zn^2+^ (480 nm)	2.5–50	Y = 9.23 × 10^−3^ X − 5.78 × 10^−3^	0.998	1.66

^1^ Y = absorbance, X = concentration of metal ion (µM). ^2^ LOD was calculated from the formula (blank + 3 SD) where blank is the response of NBD-G to zero metals, and SD is the standard deviation of the blank response [18,38].

**Table 2:** The determination of Cu and Zn in the presence of each other.

Metal Conc. (μM) in the Presence of 10 μM of the Other Metal	Found Zn^2+^ Concentration	Zn^2+^ Found %	Found Cu^2+^ Concentration	Cu^2+^ Found %
2.5	2.55	102.0	2.61	104.4
10	11.15	111.5	4.84	96.8
20	20.45	102.3	9.50	95.0
25	26.55	106.2	20.08	100.4

**Table 3:** Comparison of the performance of the proposed paper analysis with previously reported paper-based methods for the determination of Cu^2+^ ion.

Reagent	LOD (µM)	Linearity Range (µM)	Reference
Bathocuproine	25.2	78.7–1260	[43]
Polyethyleneimine	29.9	100–1000	[44]
Thioglycolic acid	33.1	100–1000	[45]
Chrome azurol S	26.8	78.7–22,047	[46]
Pyrocatechol violet	29.9	78.7–3150	[46]
NBD-G	3.9	10–200	This work

**Table 4:** Accuracy and precision study of the proposed paper-based assay for determination of Cu^2+^.

Cu^2+^ (μM)	Accuracy (Found %)	Precision (RSD %)
10	110.6	5.2
50	103.7	2.2
100	85.2	2.5
200	102.1	2.4

**Table 5:** Recovery of Cu^2+^ from rainwater samples using the test paper assay method.

Added Cu^2+^ (µM)	Recovery (%)
10	99.3
50	103.0
100	106.2
150	104.1
200	102.5

## Data Availability

Most of the data are shown in the original manuscript, and if further data are needed, it will be available upon reasonable request.

## Supplementary Materials

The following supporting information can be downloaded at https://www.mdpi.com/article/10.3390/s24237844/s1, Figure S1: Screening of the changes of the absorbance spectra of reaction products of NBD-Cl with various hydrazides or hydrazines towards different metal ions, where the tetsed probes included (a) NBD-FH, (b) NBD-AH, (c) NBD-BH, (d) NBD-DNPH, (e) NBD-TH, (f) NBD-HNH, (g) NBD-HCPI, (h) NBD-HZ, and (i) NBD-PH, where these spectra were recorded without subtraction of reagent blank.
