# Supplementary material for: The Development of a Selective Colorimetric Sensor for Cu2+ and Zn2+ in Mineral Supplement with Application of a Smartphone Paper-Based Assay of Cu2+ in Water Samples
Source: Sensors (Basel). 2024 Dec 8;24(23):7844. doi: 10.3390/s24237844 (PMC11645015; doi:10.3390/s24237844)
Supplement: Supplementary file 1 [file sensors-24-07844-s001.zip › sensors-3318692-supplementary.pdf]

Supplementary Materials

The Development of a Selective Colorimetric Sensor for Cu^{2+} and Zn^{2+} in Mineral Supplement with Application of a Smartphone Paper-Based Assay of Cu^{2+} in Water Samples

Mahmoud El-Maghrabey[1,2], Shōta Seino[1], Naoya Kishikawa[1]*, Naotaka Kuroda[1]

[1] Graduate School of Biomedical Sciences, Course of Pharmaceutical Sciences, Nagasaki University, 1-14 Bunkyo-machi, Nagasaki, 852-8521, Japan; (N. Kishikawa: kishika@nagasaki-u.ac.jp; S.S.: ssbasukeboy@yahoo.co.jp; N. Kuroda:n-kuro@nagasaki-u.ac.jp)

[2] Pharmaceutical Analytical Chemistry Department, Faculty of Pharmacy, Mansoura University, Mansoura, 35516, Egypt; (M.E. dr_m_hamed@mans.edu.eg)

***** Correspondence: Naoya Kishikawa: kishika@nagasaki-u.ac.jp

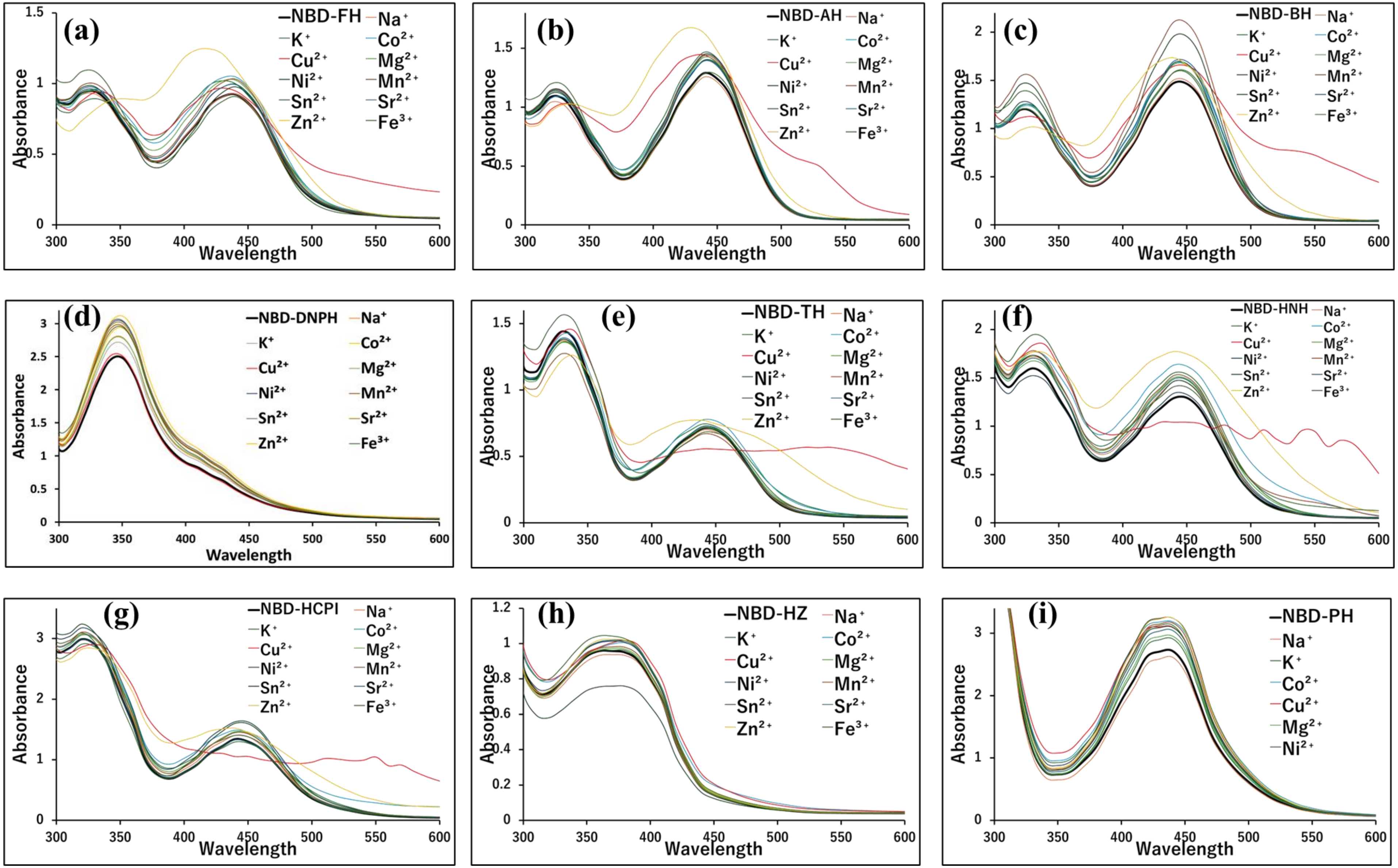

Figure S1: Screening of the changes of the absorbance spectra of reaction products of NBD-Cl with various hydrazides or hydrazines towards different metal ions, where the tetsed probes included (a) NBD-FH, (b) NBD-AH, (c) NBD-BH, (d) NBD-DNPH, (e) NBD-TH, (f) NBD-HNH, (g) NBD-HCPI, (h) NBD-HZ, and (i) NBD-PH, where these spectra were recorded without subtraction of reagent blank.
